# The Safety and Efficacy of Pregabalin Add-on Therapy in Restless Legs Syndrome Patients

**DOI:** 10.3389/fneur.2021.786408

**Published:** 2021-11-29

**Authors:** Hyoeun Bae, Yong Won Cho, Keun Tae Kim, Richard P. Allen, Christopher J. Earley

**Affiliations:** ^1^Department of Neurology, Keimyung University School of Medicine, Daegu, South Korea; ^2^Department of Neurology, Hopkins Bayview Medical Center, Johns Hopkins University, Baltimore, MD, United States

**Keywords:** restless legs syndrome, pregabalin, dopamine agonists, treatment, add-on

## Abstract

Pregabalin is increasingly being used as a first-line treatment for symptomatic control of restless legs syndrome (RLS). This study aimed to evaluate the efficacy and safety of pregabalin as add-on therapy in RLS patients already taking dopamine agonists (DA) but still in need of further management. Patients with idiopathic RLS were enrolled, and all had already been prescribed DA for at least 3 months but still had either persistent symptoms, side effects, or comorbid insomnia. An initial dose of 75 mg pregabalin was begun, adjusted as needed, and maintained at a stable dose for 4 weeks, followed by observation for a total of 8 weeks. RLS symptoms and insomnia scores were evaluated before and after add-on pregabalin treatment. Patients were monitored for side effects that could be attributed to pregabalin. A total of 32 RLS patients were enrolled, and 20 subjects remained until the endpoint. After the pregabalin add-on, the mean IRLS score showed significant improvement compared to the baseline (*p* < 0.001). The insomnia severity index score also improved (*p* = 0.036), and no serious adverse effects were observed. Our preliminary data suggests the potential for pregabalin as an add-on therapy to DA with regards to both efficacy and safety in patients who have inadequate RLS improvement.

## Introduction

Restless legs syndrome is a sensorimotor neurological disorder with a worldwide prevalence of 5–10%, and 2–3% of patients having symptoms severe enough to seek treatment (1). The discomfort and urge to move the legs, which is the prominent symptom of restless legs syndrome (RLS), occurs mainly at night. Symptomatic patients suffer from sleep disturbance and their quality of life deteriorates as a consequence (2).

Dopamine agonists (DA) are known to relieve RLS symptoms (3) and are commonly used as first-line treatment (4). Acute treatment with DA can result in side effects that may limit dose escalation and thus limit the ability of DA to fully treat the symptoms. Adverse events were documented for 79% during the first trial year of rotigotine (5), and 19–24% discontinued pramipexole by 1 year (6). Previous studies have demonstrated that despite improving symptoms, DA does not always improve sleep quality (7) and it may cause insomnia in some patients. Finally, some patients may only have partial benefits even at the acceptable, upper-dose limits. Treatment options for physicians who have to deal with these problems when trying to treat patients include moving to an alternative class of agents or add-on a second medication from an alternative class of agents. One of these alternative agents is pregabalin (6).

Pregabalin has been shown to be an effective monotherapy for managing RLS symptoms when compared to placebo or active treatment with a DA (6, 8–10). However, there have been no studies using pregabalin as an add-on agent with DA. Studies with pregabalin in RLS have reported that pregabalin is effective in improving not only RLS symptoms but also sleep structure (6, 9). Other drugs are considered second-line treatment options and have several side effects. Benzodiazepines have dependency, falling, and reduction of deep sleep issues. Opioids have many side effects such as sedation, constipation, mood change, opioid-induced respiratory depression, and dependence (11). Therefore, pregabalin shows promise as a non-dopaminergic “add-on” treatment option that might complement and compensate for some limitations with the use of single-agent DA (4, 12). The purpose of this study was to evaluate the efficacy and safety of pregabalin as add-on therapy in patients who were on DA for RLS but for whom further symptomatic improvement was needed.

## Methods

### Study Design and Participants

The study was approved by the institutional Ethics Committee of the regional hospital with written informed consent obtained from all participants. It was a prospective single-center study, conducted between January 2019 and December 2020. All patients were initially on a daily regime of DA for at least 3 months and needed additional treatment due to comorbid insomnia or persistence of symptoms despite reaching upper limits of DA dose or dose escalation was limited due to onset of side-effects.

Patients aged over 18 with idiopathic RLS symptoms were eligible for the study. RLS was diagnosed by a neurologist through a face-to-face interview based on a Korean version of the Hopkins Telephone Diagnostic Interview. Patients were required to be taking a stable dose of a DA for at least 3 months and no other RLS treatments. Enrollees in the study were required to have symptoms on at least 5 days per week or at least have 2 days of International RLS (IRLS rating scale) score over 15 per week. We evaluated for augmentation using the National Institutes of Health (NIH) criteria for augmentation (13).

The exclusion criteria were as follows: secondary RLS (e.g., peripheral neuropathy, neurodegenerative disorders, multiple sclerosis, and chronic renal failure); the presence of DA-induced augmentation; receiving any treatment that can markedly change RLS symptoms or study results, including anxiolytics or antidepressants; severe medical illness (e.g., chronic organ failure, active inflammation or infection, and congestive heart failure); any condition that causes iron deficiency (e.g., pregnancy, chronic bleeding, excluding menstruation and medically necessary phlebotomy); shift worker; untreated obstructive sleep apnea; prior experience of adverse effects to pregabalin; inability to participate in the trial; and history of suicidal thoughts or attempts within 6 months.

Baseline evaluations were conducted at the first visit. Demographic data, iron profiles, sleep questionnaires including Pittsburg sleep quality index (PSQI) (14), Insomnia severity index (ISI) (15), Beck Depression Inventory (BDI) (16), Beck Anxiety Inventory (BAI) (17), and evaluation of RLS symptoms by IRLS (18), RLS quality of life (RLS QoL) (19), Visual Analog Scale (VAS) were assessed. The questionnaires were validated in a Korean version. The initial dose of pregabalin was a single 75 mg dose in the evening, and it was adjusted up to 300 mg over 4 weeks depending on the symptoms of the patient.

Afterward, 4 weeks from the first visit, the patient was evaluated for changes in RLS symptoms and side effects of the pregabalin, and to determine a final and stable dose for the following 4 weeks.

The final visit was 8 weeks from the first visit. Again, symptom changes and adverse effects, including augmentation, were checked. In addition, the same evaluation conducted at the first visit (i.e., iron profiles, sleep scales, and RLS symptom evaluation) were repeated.

### Statistical Analysis

Clinical information, RLS symptom, and sleep status were analyzed by frequency analysis and descriptive statistics. Changes in RLS symptoms and sleep status after pregabalin treatment were analyzed using paired *t*-test. The primary variables were IRLS score and VAS. The analyses were based on an intention-to-treat model with mean substitution of imputation technique. The Pearson's correlation and multiple regression were used for the analysis of the relationship between clinical information.

## Results

**Figure 1:**
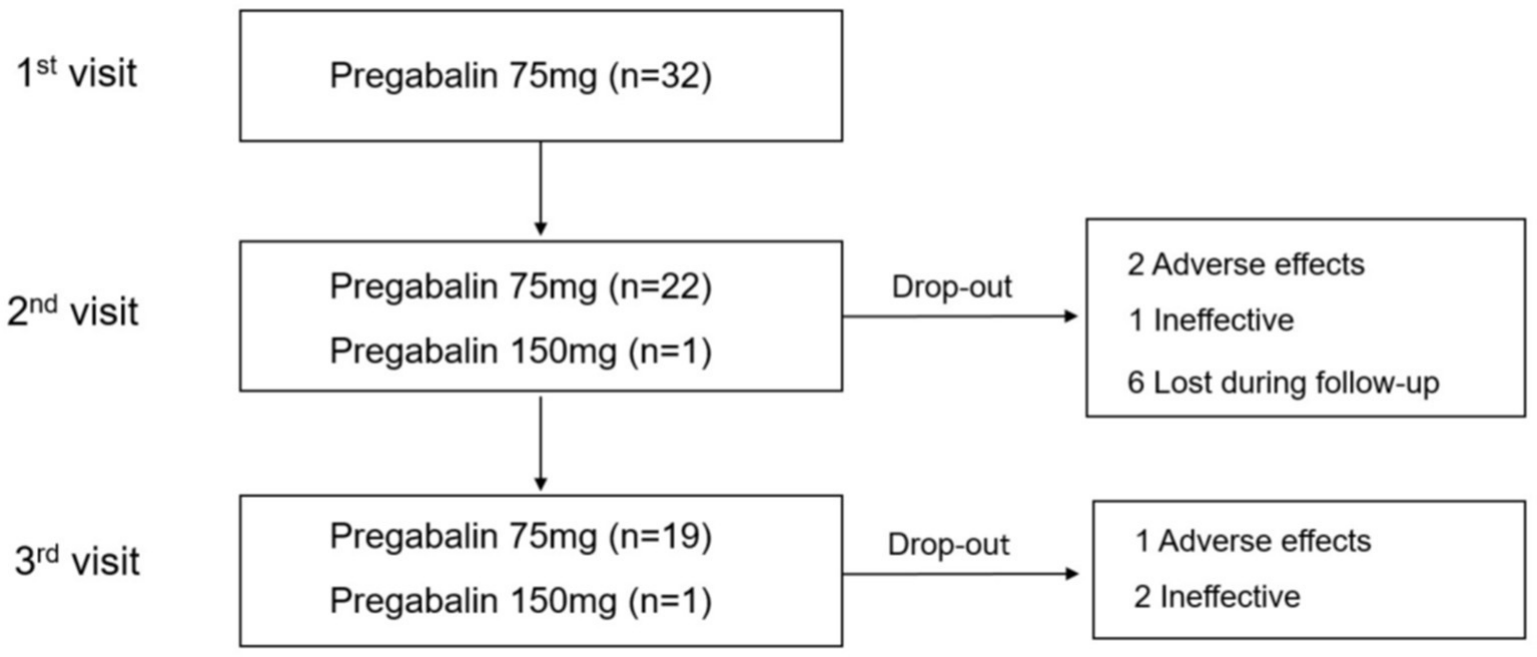
Study flow chart.

A total of 32 participants were enrolled at the first visit: six had side effects limiting increases in DA, 10 had co-morbid insomnia, and 16 had persistence of symptoms. Among the 32 participants, 12 had dropped out by Week 8. The causes of the dropouts were as follows: three were related to drug adverse effects (dizziness or sedation), two were unwilling to travel the long distance required, three were related to drug ineffectiveness, and four had personal reasons for not returning to the clinic, but unrelated to drug issues. In this group, the dose of pramipexole (*N* = 8) ranged from 0.125 to 0.75 mg and ropinirole (*N* = 4) from 0.25 to 2 mg. The median (range) treatment duration with DA was 727.08 ± 867.21 days (91–2,529 days). Additionally, 20 participants completed the Week 8 endpoint (Figure 1). The dose of pramipexole (*N* = 18) ranged from 0.125 to 0.5 mg, and ropinirole (*N* = 2) from 0.75 to 2 mg. The median (range) treatment duration with DA of the 20 participants was 472.65 ± 427.45 days (92–2,376 days). At Week 8 endpoint, 19 patients were on pregabalin 75 mg and one was on pregabalin 150 mg.

Regarding further results, 81 percent were women, mean age 59 ± 13 years. RLS duration was 12 ± 10 years with IRLS 25.7 ± 7.2, and RLS VAS 51.9 ± 32.9 when they enrolled. Moreover, 7 (21.9%) patients had clinical insomnia with ISI over 15, 15 (46.9%) were poor sleepers with PSQI over 8.5, 2 (6.3%) showed anxiety, while 10 (31.3%) had depressed moods. Hemoglobin and ferritin were within the normal range.

After adding pregabalin, RLS severity was significantly improved with mean IRLS lowered from 26.5 to 20.9 (*p* < 0.001) and the ISI showed statistically significant improvement (Table 1). None of the other scales were significantly different from the baseline. A total of six patients reported the following adverse effects on pregabalin: dizziness in two patients; sleepiness in two patients, drooling in one patient, and mild rash in one patient. Among the six patients who reported side effects, three completed the Week 8 visit.

**Table 1:** Mean (± SD) for the pre- and post-treatment RLS severity and sleep scale score.

	**At baseline**	**Post-treatment**	***p***
IRLS	26.48 ± 8.10	20.91 ± 8.66	<0.001
RLS Qol	68.63 ± 20.58	69.50 ± 22.38	0.068
RLS VAS	57.14 ± 34.81	42.38 ± 29.82	0.139
ISI	13.60 ± 7.65	12.65 ± 7.97	0.036
PSQI	11.24 ± 4.35	11.00 ± 4.59	0.093
BAI	11.10 ± 9.48	10.62 ± 9.63	0.431
BDI	13.05 ± 9.94	13.57 ± 9.53	0.330

*RLS, Restless legs syndrome; IRLS, International Restless Legs Scale; RLS Qol, RLS specific quality of life questionnaire; VAS, visual analog scale of severity; ISI, Insomnia Severity Index; PSQI, Pittsburg sleep quality index; BDI, Beck Depression Inventory; BAI, Beck Anxiety Inventory*.

## Discussion

This study showed the usefulness of pregabalin as an add-on treatment in patients who continue to have problems with RLS, despite DA use, over 8 weeks. From the perspective of RLS severity, adding pregabalin resulted in a statistically significant improvement, manifested by an improved IRLS score.

Pregabalin binds to voltage-gated calcium channels, inhibits calcium entry, and results in lowering glutamate activity and therefore decreasing excitation (20). This mechanism is hypothesized to exert an effect on RLS treatment (12). Pregabalin has several additional beneficial aspects in the management of RLS that should also be considered. Firstly, pregabalin has a positive effect on sleep (21). Current guidelines for RLS management also suggest that if patients have comorbid insomnia or sleep disturbance disproportionate to other symptoms of RLS, alpha-2-delta ligands could be first-line treatment (4). In our study, ISI was improved, indicating that pregabalin could be helpful as an add-on therapy for RLS patients with insomnia. Secondly, pregabalin is capable of controlling other comorbidities in RLS patients. As one of the alpha-2-delta ligands, it can be effective in controlling neuropathic pains and anxiety, for example (12). In addition, interaction with the BK channel has been suggested to reduce neuropathic pains (22). As regards its safety, pregabalin was well-tolerated, in line with previous studies that reported pregabalin is generally well-tolerated (9).

Generally, pregabalin is prescribed at doses ranging from 50 to 450 mg (4) and previous randomized controlled trials showed an effect in doses of over 150 mg as monotherapy (6, 9, 10). In our study, the dose of 75 mg was maintained in the majority of patients as add-on treatment. This relatively low dosage may be related to the fact that pregabalin was used as add-on therapy, not as monotherapy where dosages are routinely higher.

This study has several limitations. Our sample size was small, and the drop-out rate was moderate. The study was an unblinded, open-label study for which placebo effects could contribute to the effects we measured. Measures of sleep quality and periodic legs movement quantification during sleep on overnight polysomnography would have provided more “objective” treatment measurements.

In patients who continued to have problems with RLS symptoms despite being on a DA, pregabalin might be considered as add-on therapy to further improve RLS symptoms, thereby reducing some of the covariant insomnia problems associated with DA usage. The data supported the value of a future randomized, double-blind, placebo-controlled clinical trial in a larger population of RLS patients for whom DA are primary treatments but are not fully effective.

## Data Availability Statement

The original contributions presented in the study are included in the article/supplementary files, further inquiries can be directed to the corresponding author.

## Ethics Statement

The studies involving human participants were reviewed and approved by Keimyung University Dongsan Medical Center. The patients/participants provided their written informed consent to participate in this study.

## Author Contributions

YC and CE conceptualized research. HB and YC made contributions to the investigation. KK, YC, and CE analyzed data. HB wrote the original draft. YC, CE, and RA supervised. All authors have reviewed and edited and contributed to data curation and methodology.

## Funding

This study was supported by funding from Pharmascience Korea, Inc. Pharmascience Korea was not involved in the study design, collection, analysis, interpretation of data, the writing of this article, or the decision to submit it for publication.

## Conflict of Interest

This study received funding from Pharmascience Korea, Inc. The funder was not involved in the study design, collection, analysis, interpretation of data, the writing of this article or the decision to submit it for publication. All authors declare no other competing interests.

## Publisher's Note

All claims expressed in this article are solely those of the authors and do not necessarily represent those of their affiliated organizations, or those of the publisher, the editors and the reviewers. Any product that may be evaluated in this article, or claim that may be made by its manufacturer, is not guaranteed or endorsed by the publisher.
